# Hypoxia as a Driving Force of Pluripotent Stem Cell Reprogramming and Differentiation to Endothelial Cells

**DOI:** 10.3390/biom10121614

**Published:** 2020-11-29

**Authors:** Paulina Podkalicka, Jacek Stępniewski, Olga Mucha, Neli Kachamakova-Trojanowska, Józef Dulak, Agnieszka Łoboda

**Affiliations:** 1 Department of Medical Biotechnology, Faculty of Biochemistry, Biophysics and Biotechnology, Jagiellonian University, 30-387 Kraków, Poland; paulina.podkalicka@doctoral.uj.edu.pl (P.P.); jacek.stepniewski@uj.edu.pl (J.S.); olga.mucha@doctoral.uj.edu.pl (O.M.); jozef.dulak@uj.edu.pl (J.D.); 2 Malopolska Centre of Biotechnology, Jagiellonian University, 30-837 Kraków, Poland; neli.kachamakova-trojanowska@uj.edu.pl

**Keywords:** iPSCs, iPSC-ECs, endothelial cells, hypoxia, HIFs, CRISPR-Cas9

## Abstract

Inadequate supply of oxygen (O_2_) is a hallmark of many diseases, in particular those related to the cardiovascular system. On the other hand, tissue hypoxia is an important factor regulating (normal) embryogenesis and differentiation of stem cells at the early stages of embryonic development. In culture, hypoxic conditions may facilitate the derivation of embryonic stem cells (ESCs) and the generation of induced pluripotent stem cells (iPSCs), which may serve as a valuable tool for disease modeling. Endothelial cells (ECs), multifunctional components of vascular structures, may be obtained from iPSCs and subsequently used in various (hypoxia-related) disease models to investigate vascular dysfunctions. Although iPSC-ECs demonstrated functionality in vitro and in vivo, ongoing studies are conducted to increase the efficiency of differentiation and to establish the most productive protocols for the application of patient-derived cells in clinics. In this review, we highlight recent discoveries on the role of hypoxia in the derivation of ESCs and the generation of iPSCs. We also summarize the existing protocols of hypoxia-driven differentiation of iPSCs toward ECs and discuss their possible applications in disease modeling and treatment of hypoxia-related disorders.

## 1. Introduction

The fundamental importance of adequate oxygenation for the functioning of aerobic organisms has been recognized for centuries, and the efforts to understand the molecular mechanisms of cell sensing and adopting to oxygen availability were awarded the 2019 Nobel Prize in Physiology or Medicine, to William G. Kaelin Jr., Sir Peter J. Ratcliffe, and Gregg L. Semenza [1]. Embryonic development and the maintenance of adult homeostasis is dependent on the delivery of adequate O_2_ and nutrients to cells in the body via a functional vascular system. In contrast to atmospheric oxygen concentration (21%), its level in tissues is much lower and varies between organs. However, when it decreases below the standard values, tissue metabolism is compromised. To overcome the detrimental effects of hypoxia/ischemia the molecular machinery involving transcriptional activation of hypoxia-dependent genes, including vascular endothelial growth factor (VEGF, VEGF-A) and basic fibroblast growth factors (bFGF, FGF2) is triggered. Of importance, inadequate response to low O_2_ concentration can lead to damage in many aspects of cardiovascular development. Moreover, during the progression of various vascular diseases, the rescue mechanisms are often ineffective, and aberrations in the hypoxic response greatly contribute to serious human ischemia-related diseases.

Endothelial cells (ECs), building blood vessels, play a pivotal role in maintaining vascular homeostasis, and their improper functioning contributes to the progression of vascular-related diseases. Therefore, understanding the biology of these cells in hypoxic conditions, as well as, exploring the role of O_2_ in regulating cardiovascular events during early embryonic development may be helpful for better diagnosis and treatment. Of note, the development of human induced pluripotent stem cells (hiPSCs) [2] has opened a new era in the studies of molecular aspects of human diseases. The similarity of hiPSCs to human embryonic stem cells (hESCs), especially when (high) pluripotency potential is evaluated, makes these cells relevant to model developmental disorders. Importantly, in contrast to hESCs, the usage of hiPSCs does not raise so many ethical questions, although still some issues, particularly in the context of human reproductive cloning, or generation of human gametes, are debated [3]. Patient-specific hiPSCs may provide mechanistic insights into specific disorders, also those which are hypoxia-driven. They have other numerous biomedical applications in basic research and after directed differentiation into derivatives of all three germ layers can serve as a platform for drug testing.

## 2. Molecular Basis of Oxygen Sensing

**Figure 1:**
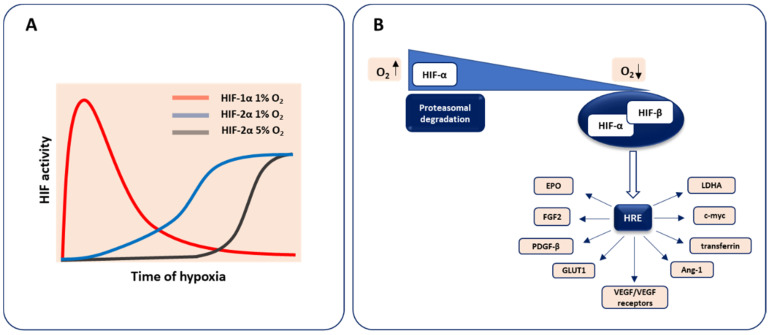
The stabilization of hypoxia-inducible factors (HIFs) under hypoxic conditions and main transcriptional targets. (**A**) HIF-1 is responsible for acute response, whereas HIF-2 mediates chronic reaction. (**B**) In normoxic conditions, the HIF-α subunit undergoes degradation pathways. When the O_2_ level decreases, HIF-α is stabilized and together with the HIF-β subunit leads to the transcriptional induction of HIF-inducible genes (mentioned in the text) via binding to the hypoxia response element (HRE) sequence.

The heterodimeric transcription factor hypoxia-inducible factor (HIF) is the pivotal regulator of molecular response to low oxygen tension. In most vertebrates, three distinct members are found. Two major isoforms, HIF-1 and HIF-2, which share some similarities but show distinct properties, including the kinetics of activation and oxygen dependence, are known to regulate oxygen homeostasis influencing embryonic development, postnatal physiology as well as disease pathogenesis [4]. It was reported that HIF-1α drives the response to acute hypoxia and decay during prolonged hypoxia, whereas HIF-2α predominantly controls the reaction to chronic hypoxia. Moreover, HIF-2α is more stable compared to HIF-1α at higher oxygen levels (5% O_2_) in neuroblastomas [5,6] (Figure 1A).

The biology of the HIF-3 factor is still not fully discovered as its multiple variants being the result of the utilization of various promoters, different transcription initiation sites, and alternative splicing exist [7].

HIF binds to DNA via N-terminal basic helix–loop–helix (bHLH) domain and activates transcription with the C-terminal transcriptional transactivation domain (TAD), whereas Per–Arnt–Sim (PAS) domain is required for the protein dimerization. This hypoxia-activated transcription factor consists of two subunits, an oxygen-sensing HIF-α, the isoform undergoing constant degradation under normal oxygen concentration through prolyl hydroxylases (PHDs)-dependent modification, and an oxygen-independent HIF-β (aryl hydrocarbon receptor nuclear translocator, Arnt), expressed at a constant level, irrespectively of oxygen status. In hypoxic conditions, HIF-α is stabilized and heterodimerizes with the HIF-β subunit to form the active HIF transcription factor able to trigger gene expression after binding to the hypoxia response element (HRE) in the promoter region of hypoxia-target genes. These HIF-regulated genes involve those implicated in cellular differentiation, metabolism, angiogenesis, etc. including VEGF and its receptors VEGFR1 (Flt1), VEGFR2 (Kdr, Flk1) as well as FGF2, platelet-derived growth factor-β (PDGF-β), erythropoietin (EPO), transferrin, glucose transporter 1 (GLUT1), lactate dehydrogenase A (LDHA), and many others (reviewed in more details in [8,9]) (Figure 1B).

## 3. Hypoxia in Early Embryonic Development and Vasculature Formation

### 3.1. Early Embryogenesis Progresses in the Hypoxic Environment

**Figure 2:**
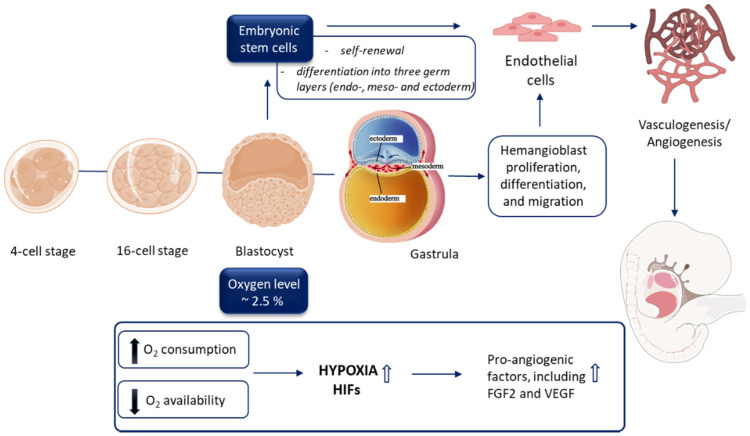
The role of hypoxia during embryonic development and vasculature formation. Embryonic development occurs in hypoxia conditions. In the embryo, a low level of O_2_ stimulates the differentiation of stem cells, including hemangioblasts (from early mesoderm), and facilitates vasculogenesis and angiogenesis through HIF-dependent mechanisms.

Embryonic development is a strictly regulated process that starts from the moment of egg cell fertilization and involves coordinated spatial and temporal changes in gene expression, cell division, migration, and cellular differentiation. Over the time of 8 weeks (duration of the embryogenesis in humans) a complex, however, not fully functioning organism grows out of a single-celled zygote [10]. Embryogenesis occurs in relatively hypoxic conditions due to the rapid cell proliferation as well as increased oxygen demand and consumption, which is not yet supported by the maternal circulation [11,12,13] (Figure 2).

Measurements performed in different mammalian species revealed that oxygen concentration in oviductal and uterine lumen varies from 1.5% (uterus of rhesus monkey) to 8.7% (e.g., rabbit oviduct and uterus) [14]. Interestingly, in hamsters and rabbits, the level of hypoxia depends on the reproductive stage decreasing significantly at the time of blastocyst formation and implementation [14]. This indicates that the pre-implemented embryo has to be adapted in vivo to low oxygen availability. In accordance, several studies confirmed the beneficial effects of hypoxia during the initial stages of mammalian blastocyst development performed in vitro. The number of goat embryos, for instance, isolated at 2- to 4-cell stage, which progressed into expanded and/or hatched blastocyst was significantly higher when cultured in 7% oxygen in comparison to normoxic counterparts [15]. A similar effect was also observed in sheep and cows. Detailed analyses of the latter species revealed that bovine embryos engage the HIF-2α transcription factor to detect and respond to hypoxic conditions. Particularly, the application of 2% oxygen resulted in the upregulation of glucose transporter 1 (GLUT1) expression indicating metabolic adaptation, when compared to 7% and 20% O_2_ concentrations. In parallel, translocation of HIF-2α into the nuclei of inner mass-forming cells was observed followed by their higher number, which could be associated with better in vivo post-transfer outcomes [16]. On the other hand, Pabon et al. reported that even short-term exposure of murine zygotes to a normoxic environment can impair their further development, whereas no changes were observed when 5% oxygen conditions were applied [17].

Due to the ethical restrictions, a detailed investigation of the environmental conditions, including the effect of hypoxia during the early stages of human embryonic development, has been limited to fertilized oocytes generated during assisted reproductive technologies (ART). Particularly, in vitro culture of such human embryos for 3 (up to 8-cell stage) to 6 (blastocyst stage) days is an intrinsic step of ART where applied conditions may strongly influence the implementation success upon in vivo transfer [18]. Indeed, Peng et al. reported that maintaining the developing zygote in 5% oxygen resulted in increased potential to form high-quality embryos and improved implementation rate in comparison to normoxic culture. However, the final pregnancy rate was not significantly different between both conditions [19]. Similarly, a meta-analysis of studies describing the application of hypoxia for ART revealed inconclusive results. Some clinical trials reported a positive effect of low oxygen concentration on the implementation. However, no differences in pregnancy rate were observed regardless of the stage of embryo transfer (either at day 2/3 or 5/6) [18]. In a large prospective randomized study in which more than one thousand in vitro fertilized oocytes were placed either in ambient or reduced oxygen level, no effect of hypoxic conditions on fertilization, embryo development, implementation, and pregnancy rate were reported. Interestingly, surplus embryos maintained at 5% O_2_ demonstrated the increased potential to reach the blastocyst stage in vitro due to the detrimental effect of normoxic conditions on the cell number on days 5–6 of development [20]. Thus, hypoxia may positively affect the preimplantation embryonic viability, but this effect is not strong enough to influence the further pregnancy rate upon in vivo transfer. Adding to the complexity of this issue, the application of in vitro method mimicking the implementation phase of human embryogenesis described by the group of Zernicka-Goetz, revealed that the hypoxic environment did not sustain the proper transition of inner cell mass of pluripotent cells (day 5–6) into epiblast phenotype (day 9–10 of embryonic development). Instead, cell death during the implementation stage was observed possibly due to increasing embryo size, leading to decreased oxygen availability inside the embryo [21].

### 3.2. ‘Physiological Hypoxia’ as a Driving Force of Vasculature Development

As indicated earlier, hypoxia is a driving force of early blastocyst development, but it also plays a key role in controlling further events during embryogenesis, including vasculature formation. In 2001, Lee et al. demonstrated, by intravenous administration of the hypoxia marker, pimonidazole, to the pregnant mouse, strongly hypoxic regions (but with the spatial and temporal existence) in developing tissues of normal embryos in vivo [22]. Of note, hypoxic areas were characterized by the colocalization of HIF-1α and VEGF. Moreover, the concomitant presence of the platelet endothelial cell adhesion molecule-1 (PECAM1) positive cells led to the later confirmed hypothesis that “physiological hypoxia” is orchestrating the development of the circulatory system that precedes all other systems [22]. As HIFs are well known transcriptional regulators of the cellular response to low oxygen levels, their involvement in vasculature development during embryogenesis has been extensively studied. Mice globally lacking *Hif1a* were shown to die by embryonic day 10.5 due to cardiovascular impairment and defects in blood vessel formation [23]. Similarly, *Hif1b* (*Arnt*^−/−^) knockout animals showed lethality around embryonic day 10.5 due to vascular abnormalities. Moreover, *Arnt*-deficient embryos demonstrated decreased VEGF mRNA and protein levels that showed the importance of the VEGF signaling during embryonic vascularization [24]. Furthermore, to confirm the role of HIFs specifically in endothelial cells transgenic mice expressing dominant-negative HIF mutant (HIFdn) only in the vasculature were created. Such animals were characterized by the inhibition of the transcriptional responses mediated by both HIF-1 and HIF-2 and died around embryonic day 11.5. In these embryos, very primitive vasculature could be detected together with severe cardiovascular defects [25]. All these examples show the important role of hypoxia on ECs proper function and development during embryo growth.

Similarly to embryogenesis, placental development is also associated with hypoxic conditions. The human placenta is formed from a stem cell population termed cytotrophoblasts which proliferate in an undifferentiated state, preferably in a hypoxic microenvironment (2.4% oxygen) than a normoxic milieu [26]. Moreover, the role of HIFs seems to be especially important in this process as placentas from *Arnt*^−/−^ and *Hif1a*^−/−^*Hif2a*^−/−^ embryos exhibit strongly defective vascularization. Moreover, in the absence of HIFs, trophoblast lineage determination is altered [27]. Importantly, the lower oxygen concentration not only initiates the development of the placenta and embryo but also protects the growing organism against the harmful effects of oxidants [28].

### 3.3. Vascular Development during Embryogenesis

The formation of the vasculature network precedes all other systems and the first sign of blood vessel formation can be observed as early as embryonic day 7.5 in the extra-embryonic blood islands of the yolk sac [29]. ECs are derived from the mesoderm [30], and during embryogenesis, the vasculature is formed via two distinctive mechanisms: vasculogenesis and angiogenesis. Vasculogenesis is the de novo assembly of the blood vessels from the endothelial precursors, known as angioblasts, and is responsible for the primary vascularization of the developing organs. Further maturation, which involves branching and sprouting of preexisting vessels, occurs via a more complex angiogenesis process [31,32]. Finally, the addition of supporting cells, like pericytes and smooth muscle cells leads to the formation of a fully functioning vascular network [33,34]. Subsequent vasculature expansion, remodeling, and specialization results in the creation of a circulatory system consisting of arterial, venous, and lymphatic vessels [35].

Over the years, many factors have been described to play a pivotal role in the process of blood vessel formation, including responsibility for the formation of angioblasts from mesenchymal cells by FGF2, pro-proliferative and pro-migratory VEGF with its receptors, and angiopoietin-1 (Ang-1), which stimulates the recruitment of the blood vessel supporting cells. Knockout of any of the mentioned factors leads to severe vessel malformation, a strong aberration of ECs function, or even death of the embryo during pregnancy [36,37,38,39,40,41]. Importantly, the expression of all of the factors can be regulated by the HIFs, suggesting that the development of a properly functioning circulatory system will depend on the subtle changes in the oxygen levels in tissues [26].

### 3.4. Endothelial Cell Origin and Differentiation

EC origin during embryogenesis was the topic of many studies. Despite the occurrence of some misleading theories, it is broadly accepted nowadays that ECs and hematopoietic cells share a common precursor, called hemangioblast [29]. Close developmental association and gene targeting studies showing that both endothelial and hematopoietic precursors are characterized by the expression of common markers, such as PECAM1, VEGFR2, CD34, stem cell leukemia (SCL), GATA-binding factor 2 (GATA2), strengthened this hypothesis [42,43].

In the beginning, the generation of the hemangioblasts from mesodermal cells occurs in the response to the bone morphogenetic protein-4 (BMP4), and by the activation of GATA2, a hematopoietic transcription factor, leading to the increased expression of VEGFR2 and SCL, two critical factors for further lineage development [44,45]. In response to VEGF signaling, hemangioblasts proliferate and, depending on the presence of appropriate factors, differentiate in either endothelial or hematopoietic progenitors. VEGF signaling is extremely important as VEGFR2 deficiency leads to the absence of proper blood vessels and embryo lethality [37]. Interestingly, the presence of the VEGFR2 at the very beginning of the hemangioblast differentiation suggests that hematopoietic cell progenitors originate from the endothelial intermediate cells, called hemogenic ECs [46]. It can be further supported by the increased expression of typical endothelial markers, such as Tie2, CD144 (VE-cadherin), and PECAM1 during the first steps of hemangioblast generation. In the course of hematopoietic cell differentiation, expression of VEGFR2 is downregulated with the simultaneous upregulation of CD45 marker, Runx-1 (AML1) transcription factor, and many others [47,48].

The main source of VEGF during embryogenesis comes from the O_2_-deprived cells, suggesting the important role of the hypoxic conditions in the differentiation of ECs. Indeed, it was shown that hypoxia leads to an increased number of hemangioblasts. Moreover, under conditions of low oxygen concentration, expression of mesodermal T-box gene Bry and BMP4 crucial for hemangioblast development was induced one day earlier than it was observed in normoxia [49].

### 3.5. Endothelial Cell Commitment and Autophagy

A low oxygen level was also described to be crucial for the further steps of EC development, meaning venous or arterial commitment. In the beginning, ECs have a default venous identity with the expression of VEGFR3 (FLT4, the receptor for VEGF-C and VEGF-D, other members of VEGF family) and downregulated arterial Notch signaling [50,51]. One of the factors responsible for the activation of the Notch pathways is VEGF. Such activation leads to the downregulation of the *Flt4* gene and the upregulation of the various arterial markers. Therefore, a low level of oxygenation, followed by HIF and VEGF activation, will promote the arterial commitment of the ECs [52]. Moreover, in 2013, the group of Wang et al. [53] demonstrated that the autophagy process is upregulated under hypoxic conditions promoting survival of the endothelial precursors *via* inhibition of apoptosis. When autophagy was inhibited using 3-methyladenine (3-MA), the hypoxic environment increased the number of apoptotic cells. Contrary, the induction of autophagy using rapamycin reduced the number of apoptotic endothelial progenitors [53].

As hypoxia seems to be one of the important factors regulating the fate of ECs during embryogenesis, it seems understandable that the modification of the oxygen level might serve as a potential modulator of ECs differentiation in vitro.

## 4. Hypoxia in the Derivation of Human Embryonic Stem Cells and Generation of induced Pluripotent Stem Cells

### 4.1. Hypoxia and hESC Culture

As the investigation of the environmental conditions during the early stages of human embryonic development is not ethically acceptable, a more detailed understanding of the mechanisms involved in this process became possible due to the development of methods for the isolation of murine and then human embryonic stem cells (mESCs and hESCs, respectively). For mouse embryos, these conditions were described independently in 1981 by Martin Evans and Matthew Kaufman as well as Gail Martin [54,55]. Cells building the inner mass of the murine blastocyst can be cultured in vitro while maintaining their pluripotency, i.e., the ability to differentiate into three germ layers (endo-, meso- and ectoderm). However, to sustain this state, a feeder cell layer is needed. It is usually composed of mitotically inactivated mouse embryonic fibroblasts (MEF), which secrete leukemia inhibitory factor (LIF) activating transcription factors crucial for mESCs growth [56]. On the other hand, these cells, deprived of appropriate in vitro culture conditions, begin the process of differentiation. Due to such properties, the ESC-based system allows for a detailed investigation of signaling pathways and transcription factors participating in various stages of specialization of different cell types including the role of hypoxia and HIFs in ECs development.

The first hESC line was isolated by Thomson et al. in 1998 [57]. Interestingly, these cells require different in vitro culture conditions than mESCs, maintaining the characteristics of stem cells in the presence of FGF2 and transforming growth factor β (TGF-β) (reviewed in: [58]). Further studies showed that at the level of gene expression profile and growth requirements, they resemble the so-called epiblast stem cells (EpiSCs) isolated from mouse epiblast at the stage of gastrulation [59]. Stimulation with FGF2 and TGF-β factors provides them with the ability to self-renew and differentiate to all three germ layers in vitro and in vivo after administration to mice with the impaired immune system - under these conditions, they form teratomas, i.e., tumors composed of ectoderm-, endoderm-, and mesoderm-derived tissues. As such, conventional hESCs have been described to demonstrate the so-called primed state (in contrast to naïve, characteristic to inner cell mass of the pre-implementation blastocyst) in which female lines undergo X chromosome inactivation (XCI) [60]. Nonetheless, Lengner et al. reported that isolation of hESCs in 5% O_2_ can inhibit this process and maintain two active X chromosomes in the cells. Further analyses revealed that oxidative stress associated with normoxic culture conditions was responsible for increased *XIST* (X-inactive specific transcript) expression inducing the XCI. Interestingly, deferoxamine, a potent stabilizer of HIF transcription factors, was described within the compounds inhibiting the detrimental effect of atmospheric oxygen concentration on XIST upregulation [60].

In accordance, the first methods of hESC culture were associated with pronounced spontaneous differentiation requiring manual picking of undifferentiated cells or constant early passaging, which sustained the proper ratio of pluripotent cells [57,61]. Appreciating the role of hypoxic conditions in mammalian pre-implementation embryonic development, Ezashi et al. assessed the effect of low oxygen concentration on hESC culture. The results indicated that maintaining the hESCs at 5% O_2_ or below significantly reduced the area of differentiated cells within the inspected colonies. A similar beneficial effect was also observed for the efficiency of embryoid bodies (EB) formation [61], genetic stability, and clonal recovery [62]. Detailed transcriptomic analysis of hESCs cultured for ten passages in 2% oxygen revealed activation of gene expression pattern characteristic for hypoxic conditions as well as pathways associated with development and differentiation [63]. Similar results were obtained for cells maintained at 4% O_2_ with a profound hypoxia-related transcriptional profile, which was involved in the control of pluripotency and hESC differentiation. Interestingly, *MYC*, encoding the c-MYC transcription factor crucial for self-renewal of pluripotent stem cells, was detected among the upregulated transcripts and its expression remained high after prolonged culture in hypoxic conditions, possibly sustaining the undifferentiated phenotype of hESCs [64]. Further analyses revealed that among HIF isoforms, HIF-2α was responsible for the activation of *MYC* transcription under low oxygen levels. Noteworthy, we have previously described a similar cross-talk between HIF-2α and c-MYC in human ECs, which is a part of the mechanism controlling interleukin 8 (IL-8) expression in hypoxia [65]. The level of IL-8, however, decreases in severe (0.5%) and mild (5% oxygen) hypoxic conditions due to the predominant negative regulation of c-MYC by HIF-1α indicating the interplay between both HIF isoforms in different cell types [65]. Interestingly, Forristal et al. added another layer of complexity to this issue, describing the HIF-3α-mediated upregulation of HIF-2α and concomitant downregulation of HIF-1α in hESCs cultured under low oxygen tension [66]. The study, on the other hand, confirmed the crucial role of HIF-2α in pluripotent stem cells as its silencing resulted in decreased expression of OCT4, SOX2, and NANOG, which constitute crucial transcription factors for maintaining the pluripotent phenotype of hESCs, together with the increased level of SSEA1, an early differentiation marker [66]. This observation was further strengthened by a recent study linking hypoxia, metabolism, and the expression of pluripotency-mediating factors. Particularly, it was reported that glycolytic flux in hESCs cultured under 5% oxygen controls the level of HIF-2α, which in turn directly upregulates C-terminal binding protein 1 and 2 (CTBP1 and 2, respectively) transcription factors promoting self-renewal of hESCs [67]. HIF-2α was also demonstrated to provide the higher activity of glycolytic flux through activation of the expression of GLUT1 in these cells [68].

Application of mESCs in which HIF-1α expression was replaced with the 2α isoform (*Hif-1α^Hif-2αKI^* model) revealed that the level of OCT4 is also regulated by HIF-2α during early embryogenesis in mouse [69,70]. Interestingly, teratomas formed by such cells were characterized by increased expression of VEGF and microvessel density, which highlighted the role of HIF-2α in vasculature formation during embryonic development [69].

### 4.2. Hypoxia and the Generation of iPSCs

Although the derivation of hESC lines provided a novel path toward the application of stem cells for investigation of human embryonic development as well as the generation of different cell types for drug screening and regenerative medicine, the research progress has been hampered by ethical concerns regarding the destruction of human embryos during hESC isolation [71]. Additionally, the availability of patient-specific hESCs was highly limited, restricting their utilization in disease modeling, while the possibility of immune rejection upon in vivo administration of hESC-derived cells raised concerns over successful outcomes of clinical trials. Thus, the breakthrough in the field occurred after the description of the somatic cell reprogramming method with defined transcription factors.

**Figure 3:**
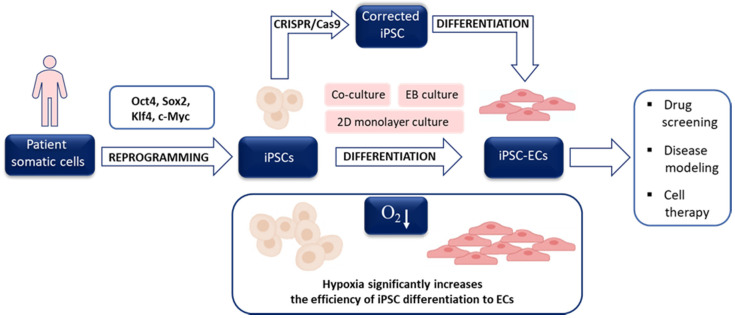
General scheme of induced pluripotent stem cells-endothelial cells (iPSC-ECs) utilization. Patient somatic cells may be used to obtain iPSC after delivery of Yamanaka’s cocktail of four transcription factors (Oct4, Sox2, Klf4, c-Myc), however, the process may be facilitated by hypoxia. After iPSC reprogramming and differentiation of iPSCs to ECs, the cells may be used in various applications. Low oxygen tension may also trigger vascular differentiation. The possibility to modify (correct) mutations in patient-specific iPSCs using CRISPR/Cas9 technology creates a unique opportunity for personalized investigations.

In 2006 Takahashi and Yamanaka demonstrated that overexpression of as little as four proteins, namely Oct4, Sox2, Klf4, and c-Myc, which regulate self-renewal and developmental potential of ESCs, in MEFs was enough to obtain a small proportion of cells with ESC-like morphology, gene expression profile, and differentiation potential. Due to the method of generation, they were called induced pluripotent stem cells (iPSCs) and could be maintained in culture in the same conditions as applied to mESCs [72]. Importantly, a year later, the same group reported that the identical set of transcription factors (called Yamanaka’s cocktail) reprogrammed human adult fibroblasts. Subsequent progress in the application of hiPSCs confirmed that the reprogramming method provides invaluable opportunities: (i) to obtain patient-specific stem cells and differentiate them into cells affected by the patient’s disease, (ii) to utilize such differentiated cells in drug testing, (iii) to generate donor-specific cells applicable in regenerative medicine and (iv) to study in vitro the mechanisms of stem cells differentiation. The iPSC-based strategy may be combined with the CRISPR/Cas9 approach to repair the mutations in specific genes allowing the unprecedented chance for personalized investigations (Figure 3). The initial efficiency of mouse iPSCs (miPSCs) and particularly human iPSCs (hiPSCs), however, was very low, which triggered the search for favorable conditions increasing the rate of successful reprogramming.

Taking into consideration the important role of physiological oxygen tension for maintaining the undifferentiated phenotype of hESCs and on the function of other stem cell populations, Yoshida et al. demonstrated as early as in 2009 that hypoxia significantly increases the efficiency of both miPSCs and hiPSCs generation).

The most profound effect was observed for the propagation of the reprogramming process in 5% O_2_ concentration. Interestingly, such conditions provided a higher rate of miPSC generation even after the transduction of MEFs with only two transcription factors—Oct4 and Klf4 [73]. The beneficial role of hypoxia on the reprogramming outcome of human fibroblasts was further confirmed by Mathieu et al., who additionally performed a detailed analysis of HIF-1α and HIF-2α activity during hiPSC development [74]. Of note, both HIF isoforms were transiently stabilized and transcriptionally active during the reprogramming process even in normoxic conditions and thus the gene expression changes observed in the transition from differentiated to pluripotent phenotype demonstrate a hypoxic signature. Interestingly, HIF-2α was indispensable for the early phase of hiPSC generation, when it mediated a switch from oxidative to glycolytic metabolism. In the later stages, its prolonged stabilization, however, significantly decreased the reprogramming efficiency due to the upregulation of TNF-related apoptosis-inducing ligand (TRAIL) and possibly downstream inhibition of caspase 3 activity. HIF-1α, on the other hand, did not exert such a biphasic effect and its stabilization was necessary to induce the metabolic switch during hiPSC generation without the negative effect on reprogramming outcome [74].

**Figure 4:**
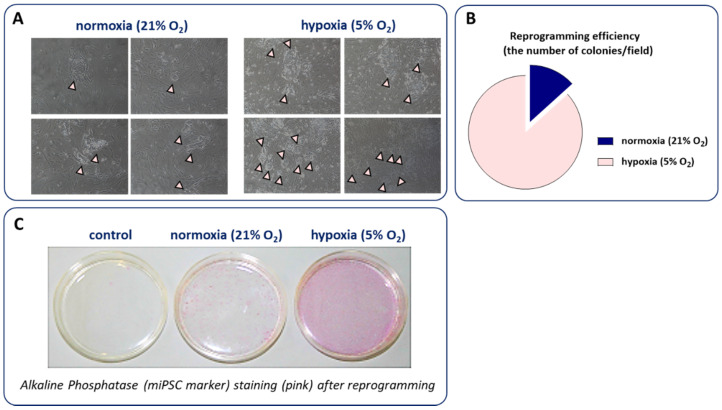
The influence of hypoxia on iPSC reprogramming. Cells kept in low oxygen concentration (5% O_2_) create more colonies (indicated by arrows) than cells cultured in atmospheric conditions (21% O_2_) as showed by (**A**) representative photos; (**B**) their calculation as well as (**C**) alkaline phosphatase (miPSC marker) staining after reprogramming.

We have also confirmed that the reprogramming of murine fibroblasts in 5% oxygen substantially increased the number of miPSCs colonies and alkaline phosphatase (miPSC marker) staining after reprogramming (Figure 4). However, due to the cost of constant hypoxia maintenance as well as the development of other approaches increasing the miPSCs and hiPSCs yield, low oxygen tension is currently not routinely used for the generation of these cells.

## 5. Hypoxia and Stem Cell Differentiation toward ECs

Over the past years, significant advances have been made in generating ECs from a variety of cellular sources and using a plethora of well-established protocols, which may differ in, among others, efficiency, cost, or duration. Differentiation of ESCs or iPSCs into ECs is mostly performed using coculture, EBs, and 2-dimensional monolayer methods (reviewed in [75]), (Figure 2). The discovery that hypoxia may trigger vascular differentiation of ESCs/iPSCs, as well as arterial specification, may result in obtaining a higher yield of therapeutically relevant cells.

### 5.1. Hypoxia Facilitates Vascular Differentiation of Pluripotent Stem Cells

Low oxygen environment, better resembling physiological conditions, not only influences the generation of pluripotent cells but also prominently modulates the differentiation toward ECs as evidenced in several in vitro studies utilizing mESCs [76,77,78], hESCs [76,79,80,81,82], hiPSCs [82,83], as well as other stem cell sources, such as glioblastoma stem-like cells [84]. Despite different experimental schemes applying also various hypoxia levels, the results unequivocally emphasize the facilitation of endothelial lineage commitment upon low oxygen concentration, mimicking the phenomenon observed during early embryonic development. Such an approach might provide the source of ECs not only for research purposes but potentially also for further therapeutic applications.

Already in 2007, the group of Chung et al. [85,86] found that the central region of hESCs-derived human EBs (hEBs) is enriched in cells that express EC markers, including PECAM1, vWF, VEGFR1, Tie2 (unlike cells growing in the outgrowth of hEBs), which can uptake acetylated low-density lipoproteins (acLDL) and exhibit increased differentiation capacity toward EC lineage. Based on these observations, the authors further hypothesized that hypoxic conditions may occur naturally in the center region of hEBs [80]. Such an assumption was strengthened by the analysis of spontaneously differentiating mouse EBs (mEBs), derived from mESCs, in which the hypoxic region was observed even under normoxic conditions, as assessed using anti-pimonidazole adduct antibody [77]. Hence, several studies investigated the impact of hypoxia as a driving force that accelerates vascular differentiation of pluripotent stem cells.

Different experimental schemes were applied to directly compare the effect of low oxygen environment (1–5% O_2_) and normoxic (21% O_2_) conditions. In general, an elevated abundance of cells expressing typical endothelial markers (e.g., CD144, PECAM1, VEGFR1, Tie2), exhibiting endothelial-like morphology (bundles of elongated cells and cobblestone area-forming cells) together with improved functionality (formation of tubule-like structures on Matrigel, uptake of acLDL) was observed upon low oxygen tension in various studies utilizing different sources of pluripotent stem cells: mESCs or mEBs, hESCs or hEBs and hiPSCs cultured with or without the addition of growth factors (summarized in Table 1). As a step forward, improved blood perfusion in ischemic hindlimb and cardiac function after myocardial infarction (MI) in vivo in mice or rats injected with ECs differentiated under hypoxic conditions was demonstrated [76,77,79].

Noteworthy, global transcriptome profiling of hESCs cultured under 1% and 5% O_2_ for 24 h, 5 days, and 15 days further confirmed changes in the expression of genes associated with e.g., vasculogenesis, angiogenesis, regulation of vascular permeability as well as vasodilatation and vasoconstriction [79]. Interestingly, White et al. [87] demonstrated that the method of ECs differentiation in which hESCs and hiPSCs were cultured and initially differentiated under hypoxic conditions (5% O_2_) yielded large numbers of ECs with limited gene expression variation between hESC- and hiPSC-derived cells. Additionally, culture under hypoxic conditions did not affect chromosomal stability [79,80], but when prolonged in extreme conditions (1% O_2_ for 7 or 15 days) resulted in significantly increased cell death [79,81].

### 5.2. Initial Exposure to Hypoxia Is Crucial for Differentiation toward ECs

Important observations were made regarding the specified hypoxia exposure period that is needed for the maintenance of differentiation toward ECs. Tsang et al. [76] tested six variations of normoxia and hypoxia (5% O_2_) period during 7 days of mESCs differentiation. It was clearly shown that a minimum of two days of initial hypoxic conditions is required, as e.g., switching to hypoxia after 2 first days in normoxia failed to increase the percentage of CD144^+^PECAM1^+^ population. In line with that was the study performed on hiPSCs differentiated for 12 days in total with the 6-day-lasting hypoxic (5% O_2_) conditions which were either initiated at the beginning (primed 5% O_2_) or at days 6–12 (secondary 5% O_2_) [82]. The expression of CD144 and PECAM1 in secondary conditions was at a similar level to normoxia, unlike in the case of primed 5% O_2_ which resulted in profound upregulation of these ECs markers [82]. Moreover, whereas the cells differentiated under hypoxic conditions during the second-half of differentiation lacked any organization, primed 5% O_2_ resulted in an endothelial-like morphology, namely elongated cell bundles and cobblestone area-forming cells with CD144^+^ cells surrounded by platelet-derived growth factor receptor beta-positive (PDGFRβ^+^) cells [82]. Hence, it might be assumed that early stages of differentiation at low oxygen tension accelerate endothelial lineage commitment.

### 5.3. The Evidence for an Arterial Phenotype of ECs Differentiated under Hypoxic Conditions

There is also evidence that ECs differentiated in hypoxic conditions exhibit rather arterial- than venous-like phenotype (aECs and vECs, respectively), similarly to embryonic development of ECs as mentioned earlier [52]. The phenotype is mostly based on the analysis of typical markers such as the evaluation of arterial (ephrin B2-*ephrinB2* and neuropilin 1-*NRP1*) and venous (ephrin type-B receptor 4-*EPHB4* and neuropilin 2-*NRP2*) markers compared to primary arterial (HUAECs) and venous (HUVECs) cells. The results revealed that the phenotype of hPSCs differentiated either for 6 or 12 days upon hypoxic (5% O_2_) conditions was more similar to HUAECs, whereas cells differentiated under normoxic conditions did not exhibit clear identity toward either phenotype [82]. In line with that Tsang et al. [76] demonstrated increased expression of not only *ephrinB2* but also *Dll4* and *Notch1* with a simultaneous decrease in the level of venous-specific CoupTFII between 3–7 days of exposure of mESCs to 1% O_2_ tension. Moreover, NOTCH1 appeared to be an important regulator of aECs maturation, consistently with literature data [52,89], as *Notch1*^−/−^ cells failed to augment the aECs phenotype but concomitantly sustained hypoxia-induced CD144^+^PECAM1^+^ population of EC progenitors [76]. The authors of the latter study emphasized the possible role of a high concentration of VEGF present in the differentiation medium on observed effects, knowing that VEGF is a critical driver of aEC fate [90].

### 5.4. The Role of VEGF in Hypoxia-Induced Differentiation toward ECs

Generally, VEGF was reported to be one of the most upregulated pro-angiogenic factors upon hypoxic conditions both on mRNA and protein (also secreted) level in the studies evaluating differentiation toward EC lineage [49,77,79,80,81]. Interestingly, to assess the importance of VEGF, Shin et al. [80] cultured hESCs-derived EBs with 50 ng VEGF for 10 days under normoxia what resulted in 1.2–3.4 times higher protein levels of VEGFR1, VEGFR2, Tie2, CD144 and PECAM1 in comparison to control hEBs. Moreover, VEGF-treated hEBs spontaneously formed vessel-like structures, similarly to hEBs kept under hypoxic conditions, and exhibited increased sprouted capillary length when cultured on Matrigel compared to control hEBs. Though it indicates that VEGF promotes vascular lineage cell development within hEBs, more direct comparison to hypoxic conditions would be needed to see if VEGF supplementation is enough to mimic or even favor the effects observed upon low oxygen environment and hence, if it could be used as an alternative approach to trigger EC commitment. A different strategy was studied by Lee et al. [77], who nicely demonstrated that treatment of mEBs with neutralizing antibodies against mouse VEGF (50 µg/mL) after 2-day-lasting hypoxic exposure decreased expression of CD144 and PECAM1. It suggests that hypoxia stimulates the EBs to produce VEGF which mediates endothelial differentiation, most probably through autocrine and/or paracrine manner. Finally, Han et al. [78] proved that extrinsic and intrinsic inhibition of VEGF attenuates, but does not abolish the development of vascular lineage cells from EBs as the result of hypoxia, pointing out that VEGF is important, but not a sole player in hypoxia-mediated angiogenic differentiation. The significance of VEGF receptors modulation in controlling the formation of the vascular network driven by low oxygen tension was emphasized as well [78].

### 5.5. HIF-1α as a Master Regulator of Hypoxia-Driven Differentiation toward ECs

As indicated earlier, the upstream regulator of VEGF, but also other angiogenic factors, is HIF-1 [8,9], with HIF-1α being potently upregulated upon differentiation of pluripotent cells in low oxygen tension [76,77,80,81].

By utilizing YC1, a HIF-1 blocker, a dose-dependent decrease of *Vegf*, *Pecam1*, *Vegfr2*, and *Cd144* in mEBs exposed to hypoxia was observed [77]. Similar results showing diminished HIF-1 target proteins and other ECs markers were obtained by the application of echinomycin, the HIF-1 transcriptional activity inhibitor [80], as well as by specific inhibition of HIF-1α using shRNA [77], emphasizing the critical role of HIF-1α in the regulation of low oxygen environment-induced vascular differentiation of pluripotent stem cells. 

Noteworthy, HIF-1α appears to be differentially regulated depending on the hypoxia exposure period. In 2008, Cameron et al. [81] were unexpectedly not able to detect HIF-1α protein in hEBs at day 16 of the differentiation under constant hypoxic conditions (1% and 5% O_2_). This puzzling result prompted the authors to apply a shorter period of hypoxia exposure, which started at day 11 of differentiation and lasted for 7 days. In that case, HIF-1α was visible already after 12 hours (unlike under normoxia), reached the peak between 1st and 2nd day and then was gradually reduced by the 5th and 7th day (in both undifferentiated hESCs and hEBs), confirming temporal HIF-1α accumulation, despite constantly low oxygen tension [82]. Concomitantly, HIF-1α target genes, *VEGF*, and *GLUT1* were increased upon the hypoxic environment (1% and 5% O_2_) as compared with 21% O_2_ at each time point [81]. A similar pattern of HIF-1α accumulation together with the upregulation of *VEGF* and *GLUT1* mRNA was observed irrespectively of the stage of the differentiation (started either at day 1, 4, 7, or 11) at which the 7-day-lasting hypoxic (1% O_2_) conditions were initiated [81]. 

More mechanistic insight into the HIF-1α regulation was provided by Tsang et al. [76]. An upregulation of HIF-1α together with its target genes, namely *Glut1*, *Pdk1*, *Pdk3*, *Pdk4*, *Ldha* during the early phase of differentiation under hypoxia (1% O_2_) was demonstrated. Of note, precise deletion of HIF-1α using CRISPR/Cas9 prominently abolished ECs differentiation as revealed by almost a 6-fold reduced percentage of a CD144^+^/PECAM1^+^ cell population in *HIF1a*-KO cells in comparison to control cells [76]. Interestingly, *Etv2*, one of the Ets transcription factors regulating ECs differentiation [91], was markedly, but temporarily elevated upon hypoxia (7-fold increase at 2–3 days and decrease at day 5 to the level comparable to normoxic conditions). HIF-1α was verified to bind to *Etv2* promoter upon low oxygen tension [76]. A deletion of *Etv2* was associated with reduced EC differentiation, similarly to the effect observed in the case of *HIF1a*-KO cells. This points out HIF-1α and Etv2 as factors required for hypoxia-mediated induction of EC progenitor differentiation and their maturation as both *HIF-1a*-KO and *Etv2*-KO cells prevented upregulation of aECs markers upon hypoxic conditions possibly through the regulation of NOTCH1 level [76].

Additionally, a comprehensive study was performed by Lee et al. [77] who applied short hypoxia exposure time and unraveled novel mechanisms driven by HIF-1α, which function is not restricted to the induction of the expression of certain genes upon hypoxia, as it may also act as a transcriptional repressor by binding to reverse HRE sequences (rHRE). Surprisingly, four such rHRE sequences specific for HIF-1α were found in the *Oct4* promoter, and HIF-1α was demonstrated to decrease Oct4 by binding to three of them [77]. Accordingly, transfection with HIF-1α (together with HIF-1β) under normoxia or knockdown by shHIF1α upon hypoxia resulted in a decrease or elevation of *Oct4*, respectively [77]. Finally, overexpression of *Oct4* (decreased upon hypoxia) attenuated expression of not only EC markers but also endodermal marker, *Troma1* (increased upon hypoxia), suggesting its negative impact on stem cell differentiation in low oxygen environment [77]. This might be particularly important in the context of controversial issues related to the influence of hypoxia on retaining stemness or promoting stem cell differentiation, what, in this particular case, indicates to be at least partially regulated by HIF-1α.

Lastly, though the effect of HIF-1β (*Arnt*) was not thoroughly studied, it should be noted that it is essential for maintaining HIF transcriptional activity. The loss of *Arnt* was shown to affect vascular differentiation of embryonic stem cells upon hypoxic conditions [78]. This indicates the integral role of both HIF-1α and HIF-1β in acquiring angiogenic features of cells in a low oxygen environment.

## 6. hiPSC-ECs in Disease Modeling

One of the most important applications of hiPSCs is to use them as a tool to better understand the mechanisms responsible for the origin and development of various diseases, but also to test the activity of potential therapeutic compounds and drug cytotoxicity in a personalized manner. Importantly, these models provide human-relevant information on disease mechanisms or drug responses, as compared to various animal models. As discussed above, hiPSCs demonstrate the capacity to efficiently differentiate into various cell types including ECs [92]. Importantly, many studies have already confirmed that patient-specific hiPSC-derived ECs recapitulate the specific pathological phenotype observed in a particular disease, either with a known genetic background or complex etiology and systemic course [93,94]. The most tractable diseases to model in vitro are those caused by mutations in single genes (monogenic diseases) [95]. The results from such studies give more relevant information, especially in case the modeling is conducted with the usage of isogenic cell lines, which differ only at the gene of interest. However, the more prevalent disorders of the vasculature, like diabetes, are caused by various factors, both genetic and environmental. Such complex diseases additionally affect different cell types and therefore are more challenging to model in vitro [75].

### 6.1. Monogenic Diseases

Pioneering cardiovascular disease modeling studies used the general comparison between hiPSC-derived ECs from healthy and diseased individuals. Such an approach was taken to study fibrodysplasia ossificans progressiva (FOP), which is a disease of heterotopic ossification caused by activating mutation in activin A type I receptor (ACVR1). Comparing hiPSC-ECs from patients with FOP to healthy control cells, the authors found increased expression of fibroblastic genes and collagen 1/2, together with increased SMAD 1/5/8 signaling upon BMP4 stimulation [96]. A similar approach was used in the modeling of Moyamoya disease (MMD), which is a slow progressive steno-occlusive condition arising in the cerebral internal carotid artery. The disease was associated with a mutation in ring finger protein 213 (RNF213), as such mutations were found in 95% of patients with MMD. Using hiPSC-ECs from MMD patients, as compared to cells derived from healthy individuals, an impairment in angiogenic response together with downregulation of cytoskeleton-related proteins was found [97]. In the study of Sa et al. [98], ECs differentiated from hiPSCs of patients with heritable or idiopathic pulmonary arterial hypertension (PAH) were compared to hiPSCs from control individuals, while observed changes were additionally confirmed in primary pulmonary arterial ECs from the same patients. Importantly, both models (primary cells and iPSCs) gave similar results with ECs from PAH patients being associated with reduced adhesion, migration, survival, and tube-formation capacity in comparison to control cells. Thus, the authors concluded that iPSC-ECs are useful surrogates and can be used to uncover novel features related to disease mechanisms [98].

**Figure 5:**
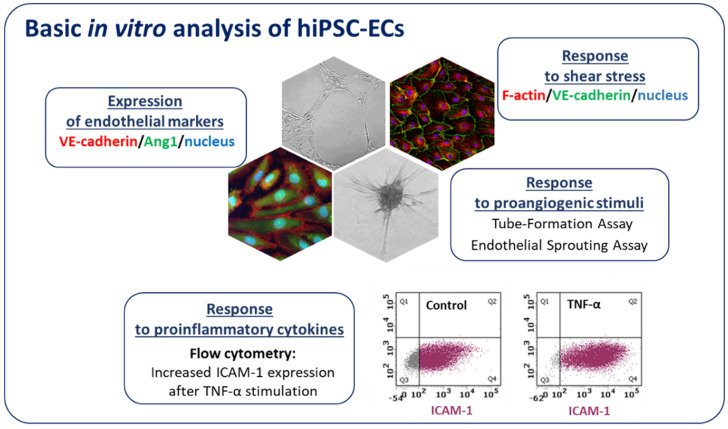
Characterization of hiPSC-ECs. Endothelial cells after differentiation from hiPSCs express endothelial markers (e.g., VE-cadherin, Angiopoietin 1-Ang1) and exert potent angiogenic capacity (they form tubule-like structures on Matrigel and spheres in endothelial sprouting assay). hiPSC-ECs properly respond to proinflammatory cytokines (the increase in ICAM-1 (intercellular adhesion molecule 1) expression is evident by flow cytometry analysis) and shear stress.

Along with the development of advanced gene-editing tools such as CRISPR/Cas9, new strategies allowing for the generation of isogenic cell lines that differ in a single gene provided a new powerful tool linking genotype to phenotype in different cardiovascular diseases. For instance, to understand the effect of bone morphogenetic protein receptor 2 (BMPR2) mutations in ECs, Gu et al. [99] used ECs differentiated from hiPSCs of patients with familial PAH (FPAH), CRISPR/Cas9 corrected FPAH, healthy individual, and unaffected BMPR2 mutation carrier. The results showed that ECs from FPAH patients are dysfunctional and with decreased survival as compared to BMPR2 corrected cells and the ones obtained from unaffected mutation carriers. This suggests that additional BMPR2 modifiers play a role in the protection of unaffected carriers from FPAH phenotype [99]. Moreover, using FPAH iPSC-ECs carrying BMPR2 mutation and CRISPR/Cas9 corrected cells, the authors were able to show the importance of the noncanonical p38 signaling pathway in response to BMP4 [99]. Looking for the disease mechanisms of calcific aortic valve disease, which is the second most prevalent cause for heart surgery, iPSCs isogenic cell lines were used. Comparing hiPSC-ECs from patients to the respective corrected isogenic lines, the epigenetic mechanism of NOTCH1 haploinsufficiency on activation of osteogenesis gene expression and inflammatory gene networks was found [100]. In a different study, CRISPR/Cas9 targeted mutation was used to prove the disease phenotype suspected to be caused by the cardiac-specific transcription factor GATA4 in patients with bicuspid aortic valve (BAV), a heritable congenital heart disease. GATA4 was identified as a potential target while surveying the genome of such patients, and its mutation was associated with impaired endothelial-to-mesenchymal transition, a process important for normal aortic valve formation [101]. Similarly, in our studies using hiPSCs isogenic lines generated through the introduction of mutation in hepatocyte nuclear factor 1A (*HNF1A*), we aimed to model the endothelial state of patients with maturity onset diabetes of the young (*HNF1A*-MODY) [92]. In our hands, hiPSC-ECs expressed typical endothelial markers, exerted angiogenic functionality, properly responded to inflammatory cytokines as well as shear stress (Figure 5). Vascular complications are quite common in this MODY subtype and we showed that hiPSC-derived ECs with a monoallelic mutation in the *HNF1A* gene, as occurring in *HNF1A*-MODY patients, have increased vascular permeability in comparison to ECs derived from the isogenic control line. The effect was even stronger in line with biallelic mutation of the gene, suggesting a certain predisposition of *HNF1A*-MODY patients to microvascular complications [92]. Currently, we are also using hiPSC-ECs to better understand the dysregulation of angiogenesis in Duchenne muscular dystrophy (DMD). Using the CRISPR/Cas9 method we established an isogenic cell line with deleted *DMD* exon 50, resulting in a complete absence of dystrophin, a mutation frequently found in DMD patients. Our ongoing experiments indicate that hiPSC-ECs are a great tool to study the angiogenesis-related changes in the pathology of DMD.

### 6.2. Complex Vascular Disease—Diabetes

Although attempts have been made to model the effects of metabolic diseases using hiPSCs from diabetic patients, no studies have explored the effect of diabetes on ECs [102]. Diabetes is a group of metabolic diseases that affects over 300 million people worldwide [103]. However, the cause of the disease is multifactorial, including both environmental and genetic factors. A great part of the morbidity and mortality associated with diabetes results from chronic vascular complications. Even though hyperglycemia was considered as the main causative for diabetes-related endothelial dysfunction, recent studies show that vascular complications can still occur in patients independent of the diabetes duration and their glycemic status [104]. For instance, Drawnel and colleagues showed that culturing cardiomyocytes derived from control hiPSCs under hyperglycemic conditions led to the development of features characteristic for diabetic cardiomyopathy, in particular, hypertrophy, dysregulation of sarcomeres, abnormal electrophysiological, and metabolic properties as well as increased oxidative stress [94]. Importantly, the same phenotype was displayed by cells obtained from type 2 diabetes (T2D) patients even under normoglycemic conditions, and the extent of pathophysiological changes correlated with the advancement of the disease [94]. Currently, limited studies have addressed the issue of endothelial dysfunction in relation to diabetes, using various approaches that limit the number of possible causative factors. Knowing that AKT2 kinase (PKB) is an important mediator of insulin signaling and its disturbance leads to early onset diabetes and obesity, Roudnicky et al. used ECs derived from hiPSCs with dysregulated AKT2, to model the endothelial dysfunction [102]. The results showed that such cells have an increased proinflammatory response, which may further contribute to coronary artery disease [102]. In a different study, RNA-binding protein Quaking-7 (QKI-7) was found to be upregulated in iPSC-ECs after exposure to hyperglycemia and in hiPSC-ECs from diabetic patients. QKI-7 upregulation was correlated with disrupted cell barrier, enhanced monocyte adhesion, and compromised angiogenesis, suggesting this protein as a possible new strategy for the treatment of diabetic vascular complications [105]. Another approach could be the use of information from genome-wide association studies (GWAS), which have successfully identified a large number of genetic loci associated with risks of complex traits in cardiovascular diseases. Recently, at least 57 independent loci within the human genome were identified as risk factors for developing type 1 diabetes (T1D) [106]. However, it is not possible to study one gene at a time without incurring significant epistatic effects from other risk genes. The likelihood of finding two individuals differing at only one risk gene, while having identical variants at the remaining 56 risk regions is infinitely small. Therefore, the additional downstream analysis should be performed to narrow the list of potential risk factors. In addition to the number of variants and overlapping pathways, there are additional layers of complexity at the cellular level. To begin to address these challenges, the researchers need robust platforms to study the effects of individual risk alleles in various cell types under controlled conditions, which could be achieved through a hiPSCs-based approach [106]. Overall, there are encouraging reports suggesting that this complex metabolic syndrome can be effectively modeled with hiPSCs technology. However, there is a current lack of information, whether these in vitro models can fully reproduce dysfunctional phenotype through hiPSC-derived ECs [107]. 

## 7. Application of hiPSC-ECs for Ischemia-Related Disorders

**Figure 6:**
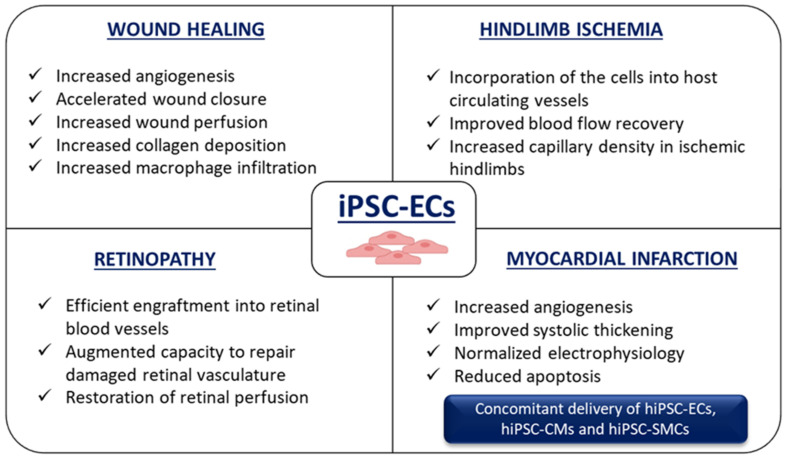
Possible applications of hiPSC-ECs in hypoxia-related disorders. hiPSC-ECs may serve as a valuable tool to study mechanisms of wound healing, retinopathy, hindlimb ischemia, and myocardial infarction (see details in the text).

As indicated above, iPSC-ECs can function as an excellent tool for disease modeling and can be used in pathophysiological studies to model endothelium-related diseases. The pathogenesis of vascular diseases is associated with the altered functioning of not only ECs but also other cells building blood vessels, like vascular smooth muscle cells (VSMCs). They can be both derived from iPSCs and applied to mimic vascular abnormalities to better understand the molecular mechanisms underlying human diseases. hiPSC-ECs are highly relevant in both disease modeling and therapeutic interventions with their beneficial potential already tested in many preclinical animal studies resembling the conditions associated with hypoxia, such as wound healing [108,109,110,111], hindlimb ischemia [86,110,111,112,113,114,115,116], retinopathy [117,118,119], and myocardial infarction [120,121,122,123] (Figure 6). In these studies, it was demonstrated that the transplantation of hiPSC-ECs improved the disease condition through either integration into the host vasculature or paracrine activation.

### 7.1. Wound Healing

Angiogenesis is an essential component of the normal wound healing process, as it restores perfusion to damaged tissues and provides key nutrients. On the other hand, the proper vascularization is reduced in the hypoxic environment of chronic wounds. Several studies utilized iPSC-ECs in both non-ischemic and ischemic wound healing, indicating their role in the facilitation of the blood vessel formation and perfusion to injured tissues. Moreover, the usage of hiPSC-ECs may be a promising strategy for the treatment of diabetic wounds, in which the low oxygen environment greatly contributes to observed complications [108,109,110,111].

In the above-mentioned interventions, hiPSC-ECs were delivered mostly by intradermal injection or topical application. In a murine model of wound healing, improved blood perfusion in wounds, and increased vessel density was observed after hiPSC-ECs treatment [109]. The abundance of PECAM1-positive vessels was confirmed in wounds at both 7 and 14 days after the topical application of 5 × 10^5^ hiPSC-ECs. The expression of different angiogenic markers was significantly increased, and the collagen content was also prominently higher after hiPSC-EC delivery. The calculation of wound closure in the following days after cell application revealed statistically significant improvement at day 4 and 10 post-injury and hiPSC-EC-treated wounds achieved complete closure 4 days earlier than in control conditions [109].

In a different approach, Kim et al. [108] tested concomitant delivery of 6 × 10^4^ hiPSC-ECs and 4 × 10^4^ hiPSC-SMCs. Such combinatory treatment was quite effective, and increased angiogenesis prominently supported wound closure. The comparison of the angiogenic secretome of hiPSC-ECs and HUVECs showed greater release of VEGF, FGF-4, and epidermal growth factor (EGF) by ECs differentiated from hiPSCs. Consistent with in vitro data, enhanced in vivo neovascularization in groups treated with hiPSCs-derived ECs and SMCs in comparison to HUVECs mixed with human vascular SMCs (HVSMCs) was observed. Moreover, the co-implantation of hiPSC-ECs and hiPSC-SMCs gave better results in terms of vascular perfusion and arteriole density compared with mice treated with hiPSC-ECs alone [108].

Another option to increase the effectiveness of hiPSC-ECs delivery to the ischemic milieu and overcome the problem with the low rate of in vivo cell survival might depend on the usage of special biomaterials/scaffolds. Culturing of hiPSC-ECs on electrospun polycaprolactone (PCL)/gelatin scaffold (70:30) for seven days and then implanting cells in vivo to wild-type FVB/n mice improved cell survival up to 3 days, whereas the engraftment of the hiPSC-ECs without any modifications was very poor, and their viability dramatically decreased already 1 hour after injection [110]. The enhanced recruitment of macrophages in the case of scaffold-assisted hiPSC-ECs delivery was also evident. Of note, a similar number of cells as in the above-mentioned studies (~3 × 10^5^) was transplanted into severe combined immunodeficient (SCID) mice (5 × 10^5^) [109], in which quite good efficacy of cells without any biomaterials was described. These differences underline enhanced cell engraftment in SCID mice in comparison to mice with a properly working immune system, but still poor cell survival represents a major limitation to the long-term therapy.

Another example of supporting material used to enhance hiPSC-ECs bioavailability can be acrylated hyaluronic acid, which was shown to promote the delivery of hiPSC-ECs in a model of diabetic wounds in mice [111]. Although it is well established that biomaterials exert beneficial effects on hiPSC-ECs delivery, it has to be taken into consideration that the technical parameters of the scaffolds have to be determined carefully, as e.g., the diameter and alignment of nanofibrillar collagen scaffolds may affect the properties of hiPSC-ECs (as it was shown that biomaterials greatly influence EC properties in normal and hypoxic conditions [124]). Taking all into account, hiPSC-ECs provide a great therapeutic option for the treatment of (hypoxic) wounds, but the optimal delivery and the enhancement of their in vivo survival in chronic wounds by the use of the appropriate biomaterials warrants further research.

### 7.2. Hindlimb Ischemia

Critical limb ischemia (CLI) is an advanced stage of peripheral arterial disease (PAD) manifested by chronic ischemic pain at rest, ulcerations, or limb necrosis [125]. The therapeutic potential of hESC-ECs/hiPSC-ECs has been demonstrated using animal models of CLI (mostly hindlimb ischemia model—HLI, obtained by femoral artery ligation [126]), in which the ischemic tissue has to deal with insufficient oxygen and nutrient supply.

Intramuscular injection of hESC-ECs into ischemic limbs one day after surgical induction of HLI in athymic mice resulted in accelerated capillary and arteriole densities and increased blood perfusion [86]. Of importance, the functional effect of this therapy was evident, as the improvement of ischemic limb salvage by hESC-EC transplantation was demonstrated—in the group of medium-treated animals, 9/10 mice lost the limb, whereas after cell therapy, limb loss was seen only in 3 out of 11 animals. The beneficial outcome of hESC-ECs in the ischemic leg was mediated through the paracrine effect and increased production of the pro-angiogenic factors (VEGF, Ang1, FGF2) together with the direct incorporation of transplanted cells into vascular structures. This led to the functional recovery of ischemic tissue and normalization of the pathological vascularization [86]. Of note, the favorable result (enhanced reperfusion in the ischemic limb) was demonstrated for hESC-ECs and hiPSC-ECs in comparison to HUVECs [116].

Lee et al. [113] showed that the combination of hiPSC-ECs with appropriate biomaterials may increase the efficacy of cell therapy also in chronic HLI. Although blood flow was significantly improved in mice after femoral artery ligation receiving either hiPSC-ECs or hiPSC-encapsulated in special nanomatrix gel (in comparison to medium-, biomaterial-, HUVECs- and HUVECs/biomaterial-treated animals), the quantitative analysis of vascular density in hindlimb muscles revealed the superiority of biomaterial-enhanced hiPSC-ECs delivery. The statistically significant difference in cell engraftment of ischemic tissues was found for hiPSC-ECs encapsulated within the nanomatrix gel in comparison to non-facilitated cell delivery. A similar study was performed by Foster et al. [114], who used the recombinant hydrogel, termed SHIELD (Shear-thinning Hydrogel for Injectable Encapsulation and Long-term Delivery) to increase cell survival and therapeutic efficacy of hiPSC-ECs in the murine HLI model of PAD.

The therapeutic potential of hiPSC-ECs might be related to the appropriate way of cell delivery. However, the comparison of intramuscular (IM), intra-femoral artery (IA), or intra-femoral vein (IV) injections of mESC-ECs (5 × 10^4^ cells for IM and IA as well as 5 × 10^5^ cells for IV route) in a syngeneic model of HLI confirmed the engraftment of mESC-ECs into the limb vasculature after 2 weeks as well as improved limb perfusion and neovascularization regardless of route of administration [115].

In summary, iPSC-ECs therapy for limb ischemia was shown to be safe, well-tolerated, and effective. However, the increased survival and enhanced engraftment of cells in vivo was noted after biomaterial-facilitated delivery.

### 7.3. Retinopathy

Retinal vascular abnormalities, like diabetic retinopathy and retinal vein occlusion, are associated with retinal ischemia and degeneration, resulting in vision loss. Cell therapy aiming at the restoration of the function of both damaged retinal vasculature and neurons was tested in several studies [117,118,119].

Park et al. [117] found that hiPSC-derived CD31^+^CD146^+^ vascular progenitors obtained from cord blood were effective in homing and integrating into injured retinal capillaries. The incorporation into damaged vessels lasted up to 45 days, and the repairment of the non-functional vasculature was therefore achieved. In contrast, vascular progenitors derived from iPSCs obtained from fibroblasts did not exert such capabilities [117]. In contrast, in the oxygen-induced retinopathy (OIR) in C57/BL6 neonatal pups, prominent angiogenic response and reduction of the post-injury avascular area were also observed in the retinas after intravitreal injection of 1 × 10^5^ iPSC-derived ECs with properties of the cord-blood endothelial colony-forming cells (ECFC) [118].

In a very recent paper by Cho et al. [119], the comparison of the angiogenic potential of hiPSC-ECs with mature human retinal endothelial cells (HRECs) in response to hypoxia was performed. In the mouse model of OIR, only hiPSC-EC colocalized with host vessels, and this effect was dependent on the stromal cell-derived factor-1α (SDF-1α)/CXCR4 axis. Interestingly, the procedure was performed not only in immunocompetent C57BL/6 but also in immunodeficient NOD/SCID mice, and the authors reported a similar superior effect of colocalization, which did not depend on the mouse strain/immune system. It should be noted that the possible immunogenic effect of ECs delivery to immunocompetent mice (not subjected to immunosuppression) was not taken into consideration in this study, which is an important issue when such experiments are conducted [127].

As relatively little attention has been given to the use of hiPSC-derived cells in ischemic retinal conditions, further research is needed to confirm its utility in the restoration of retinal perfusion and retinal vascular regeneration attenuating ischemic damage.

### 7.4. Myocardial Infarction

Cardiovascular diseases are the leading cause of death worldwide and new therapies targeting diseased hearts are undeniably needed. iPSC technology offers a range of possibilities. In the mammalian heart, cardiomyocytes (CMs), ECs, fibroblasts (FBs), and perivascular cells are essential for normal heart homeostasis. Depending on the method of the estimation, the relative ratios of these cell subtypes greatly vary and may be influenced by numerous conditions, including age [128,129]. Nevertheless, in terms of myocardial infarction (MI), trials are mostly conducted with CMs (including hiPSC-CMs) but the combined stem cell therapy, with concomitant delivery of hiPSC-ECs and hiPSC-SMCs, has the greater potential to form stabilized functional vessels providing long-lasting support to the patients’ blood vessel system [121]. Besides, hiPSC-derived cells of multiple cardiovascular lineages mixed with biomaterials allowing physical support for cells and being the origin of factors enhancing differentiation and/or functional maturation of hiPSC were tested in animal models. As mentioned earlier, the various components/properties of such biomaterials may greatly affect the biocompatibility with different cell types/conditions.

After studies with canine-derived iPSC-ECs in an immunodeficient mouse model of MI showing short-term improvement in myocardial contractility [130], human cells were also investigated in various experimental settings. Prado-Lopez et al. [79] utilized endothelial-like cells generated from hESCs differentiated in hypoxia (as discussed earlier) to check their functionality in a rodent model of acute MI. After induction of MI, rats were randomized to receive saline or 3 × 10^5^ hESC-ECs per animal (injected at five distinct points of the infarct border zone). Echocardiograph parameters measured after 2 and 4 weeks after transplant, as well as histological analysis, showed functional heart recovery, decreased infarct size, and a lower rate of fibrosis in animals receiving hESC-ECs in comparison to saline-treated rats [79].

In a porcine model of postinfarction left ventricular (LV) remodeling, Xiong et al. [120] tested delivery of combined transplantation of hESC-ECs and hESC-SMCs with a fibrin three-dimensional (3D) porous scaffold biomatrix. Significant engraftment of hESC-derived cells resulted in the functional benefits: increased neovascularization, LV function improvement, and reduction of infarct size that occurred predominantly after fibrin patch enhanced delivery. The additional modification was done by Ye at al., as a 3D fibrin patch supplemented with insulin-like growth factor (IGF-1, the cytoprotective agent, which can improve cell survival rate) was used for intramyocardial transplantation of hiPSC-derived CMs, ECs, and SMCs (2 × 10^6^ of each cell type, 6 × 10^6^ cells in total) in the porcine model of ischemia-reperfusion (IR) injury [121]. Such tri-lineage, IGF-1-containing fibrin patch-enhanced cell transplantation resulted in the abundance of PECAM1^+^ structures, improved LV function, decreased infarct size, and apoptosis. Moreover, the development of new technologies offered delivery of various cardiovascular cell populations (including iPSC-ECs) using a cell sheet technology what resulted in the creation of so-called cardiac tissue sheets (CTSs), which were successfully applied in rat models of MI with mouse ESC- [122] and human iPSC-derived cells [123].

In summary, the application of hiPSC-ECs offers a unique opportunity for the treatment of hypoxia-related disorders but still, this method is considered innovative and more studies, both in animals and finally in humans, remain to be performed.

## 8. Limitations and Future Perspectives

Although hiPSC-ECs are suggested to be used as a tool to study patient-specific vascular diseases, their application for personalized medicine is still limited. iPSC differentiation protocols to ECs are not fully optimized, and various schemes are proposed, also in the context of hypoxia application to increase the differentiation efficiency. The variability in the obtained cells may be related to the existence of heterogeneous populations in differentiated iPSC-ECs with a relatively low number of bona fide ECs. Paik et al. [131] have found that during differentiation, in addition to iPSC-ECs expressing endothelial-specific genes, the non-endothelial cell types of a mesodermal lineage, e.g., cardiomyocyte-like cells, hepatic-like cells, and vascular smooth muscle cells are generated. Moreover, using droplet-based single-cell RNA-sequencing (scRNA-seq), the authors identified four major (distinct) subpopulations, namely metabolically active, immune-responsive, arterial-specific, and activated ECs which were enriched in Claudin-5 (CLDN5), Apelin Receptor (APLNR), Gap Junction Protein Alpha 5 (GJA5), and Endocan (ESM1) genes, respectively. Moreover, the issue of direct specification toward arterial, venous, and lymphatic phenotype was addressed in several studies [132,133]. Uenishi et al. described NOTCH-mediated specification of iPSCs to arterial-type endothelium [132] whereas D’Souza et al. have found that differentiated CD144^+^CD31^+^CD34^+^ ECs are heterogeneous and include three major subsets with not only distinct hematogenic properties but also various capacities to give rise to arterial, venous, and lymphatic lineages [133]. Of note, not only differences related to the vessel type should be taken into consideration during iPSC-ECs studies, as the functional heterogeneity depending on the tissue in which ECs reside are also important. The detailed analysis of the data from tissue-specific mouse ECs generated by the *Tabula Muris* consortium [134] identified genes and molecular pathways that govern the organ-specific role of ECs. Moreover, it was observed that the subsets of ECs within certain tissues are sex-specific [135]. These results may be helpful in the future analysis of the diversity of human ECs and utilization of hiPSC-ECs in targeted, tissue-specific drug testing and studies of sex-specific cardiovascular disease models and treatments.

Lastly, it is worth mentioning ECFC or “endothelial outgrowth cells” with well-defined characteristics and intrinsic angiogenic potential, which are now considered as true endothelial progenitors that can be obtained from numerous sources, ranging from the cord and peripheral blood to lungs and placenta [136,137,138]. Importantly, a growing body of evidence suggests the therapeutic potential of ECFC in, amongst others, hematological disorders, ischemic heart disease, and diabetes (recently reviewed in: [139,140]). In the context of iPSCs, as mentioned earlier in the text, the group of Prasain et al. described a protocol for differentiation of human iPSCs to cells similar to ECFC, which were able to repair mouse ischemic retina and limb [118]. It would be of particular interest to study if the effects of iPSC-derived ECFC infusions mimic or even outperform ECFC obtained with the standard methods, which creates a novel, unexplored so far research area with great clinical applications.

## 9. Conclusions

hiPSC-ECs are a promising source of cells that may be used to investigate vascular development and endothelial function. Their role in both disease studies and therapeutic interventions is beyond doubt, but the future work should concentrate on the development of the appropriate, organ-specific vascular cell differentiation protocols that have to be applied to obtain arterial, venous, and lymphatic specifications. These will help to realize the full potential of hiPSC-ECs in precision medicine and hypoxia-driven cardiovascular disorders.

## Figures and Tables

**Table 1:** The summary of studies investigating the impact of hypoxia on angiogenesis-related markers and properties of pluripotent stem cells.

Cell Type	Hypoxia Level	Duration ^#^	Scheme of the Experiment	Angiogenesis-Related Markers and/or Properties Increased vs. Normoxia (20% or 21% O_2_)	References
**mESCs**	1% O_2_	7 days	Undifferentiated mESCs plated on collagen IV-coated plate and differentiated in ECDM containing Iscove’s Modified Dulbecco’s Medium (50%), 50% Ham’s F-12 medium (50%), supplemented with N2, B27 retinoic acid, 0.05% lipid-rich BSA, ascorbic acid (50 ng/mL), MTG (50 ng/mL), human VEGF (50 ng/mL), human FGF2 (10 ng/mL), and BMP4 (2 ng/mL)	CD144, PECAM1 mRNA and protein Percentage of CD144^+^PECAM1^+^ cell population HIF-1α protein *Glut1*, *Pdk1*, *Pdk3*, *Pdk4*, *Ldha*, *Etv2*, *Ephb2*, *Notch1*, *Dll4* mRNA	[76]
**hESCs**	1% O_2_	7 days	Undifferentiated hESCs plated on Matrigel-coated plate in Essential 8 medium. The next day, medium replaced with ECDM containing Iscove’s Modified Dulbecco’s Medium (50%), Ham’s F-12 medium (50%), insulin-transferrin-selenium-X, chemically defined lipid concentrate (1%), BSA (5 mg/mL), ascorbic acid (50 ng/mL), and 1-Thioglycerol (200 μM) supplemented with activin A (25ng/mL), BMP4 (10 ng/mL), VEGF (50 ng/mL), CHIR (1.5 μM) and incubated for 3 days. At days 3, 5, 7 medium replaced with serum-free differentiation medium supplemented with VEGF (50 ng/mL) and SB431542 (10 μM)	Percentage of CD144^+^PECAM1^+^ cell population	
**hESCs**	1% and/or 5% O_2_	up to 15 days	Undifferentiated hESCs cultured on ECM from human foreskin fibroblasts under hypoxic conditions in a foreskin-fibroblast conditioned medium composed of DMEM/F12, BME (100 µM), L-glutamine (1 mM), NEAA (100 mM), serum replacement (20%) without changing the media	Endothelial-like morphology of the cells *VEGF*, *VEGFB*, *VEGFR2* mRNA ANGPTL4 mRNA and protein CD34 mRNA and protein, CD34^+^ cell population ECs phenotype: * CD34^+^ population: 57.7% VEGF^+^ population: 53.4% ANGPTL4^+^ population: 80.8% PECAM1^+^ population: 11.6% vWF^+^ population: 31.9%	[79]
			As above but after 5–7 days cells plated on Matrigel and further cultured for 1–7 days	Sprouting of cellular aggregates expressing CD34 (24 h) Formation of cordlike structures expressing CD34 and VEGFR2 (3 days) More dense cordlike structures expressing PECAM1 and vWF (7 days)	
**mEBs** **Derived from mESCs**	1% O_2_	3 days	After 3 days from mEBs formation, mEBs were kept under hypoxic conditions for 3 days for spontaneous differentiation	*Vegf*, *Vegfr2*, *Pecam1*, *Acta2*, *Cd144*, *Vegfr1*, *Fgf2* mRNA HIF-1α, HIF-2α protein	[77]
		2 days	After 3 days from mEBs formation, mEBs were kept under hypoxic conditions for 2 days and seeded on a plate coated with 0.3% gelatin in DMEM/10% FBS. Next day, the culture medium was replaced with EGM2 medium supplemented with FBS (5%) and cells were incubated up to 14 days	Spreading of mEBs PECAM1^+^, CD144^+^ cells HIF-1α protein *Vegf*, *Pecam1*, *Vegfr2*, *Cd144* mRNA Junctional distribution of PECAM1 exhibiting tubular and branching structure especially in the central region on 7 and 14 days after reattachment α-SMA, CD144 positive cells in the outgrowth region on later time points, 14 days after reattachment	
**mEBs** **Derived from mESCs**	3% O_2_	5 to 10 days	mEBs differentiated under hypoxic conditions in methylcellulose or in suspension for 5 to 9 days without exogenous VEGF	PECAM1^+^ cells	[78]
			mEBs differentiated for 7 and 10 days	*Adm*, *Ang1*, *Ang2*, *Vegfr2*, *Tie2*, *Tie1* mRNA (by day 7) *Epo*, *Tie2*, *Tie1* mRNA (at day 10)	
			Differentiation of mESCs into 10-day EBs in methylcellulose containing VEGF (25 ng/mL) and FGF2 (100 ng/mL). EBs were then replated in collagen-type-I gel matrix for 4 days	Increased percentage of highly angiogenic, sprouting cells	
			As above but with a lower concentration of FGF2 (25 ng/mL) and VEGF (5 ng/mL or without this factor)	Elevated number of highly angiogenic, sprouting cells	
			EBs differentiated in suspension or methylcellulose cultures in the absence of exogenous VEGF under hypoxic conditions up to 9 days	VEGFR2^+^mVEGFR1^+^ cells sVEGFR1 protein	
**hEBs** **Derived from hESCs**	3% O_2_	7 days	hEBs transferred to hypoxic conditions 3 days after hEBs formation (cultured in DMEM/F12 medium supplemented with serum replacement (10%), L-glutamine (1 mM), NEAA (1%), BME (100 mM) without FGF2 treatment	*VEGFR2*, *PECAM1*, *CD144*, *TIE2*, *FGFR1*, *PDGFBR* mRNA HIF-1α, VEGF, FGF2, ANG1, PDGFB/PDGF-BB on mRNA and protein VEGF, FGF2, PDGF-BB and, to a lesser extent ANG1 on protein (secreted) Percentage of VEGFR1^+^, TIE2^+^, VEGFR2^+^, CD144^+^ and PECAM1^+^ cell populations	[80]
			Cells plated on Matrigel after 7 days for additional 3 days	PECAM1^+^ and vWF^+^ cells spontaneously forming vessel-like structures Increased number of sprouts and, to a lesser extent, their length	
**hEBs** **Derived from hESCs**	1% and 5% O_2_	7 days	Differentiating hEBs exposed to hypoxic conditions in a sealed 6.2-L modulator incubator; half-media changes occurred every 3 to 4 days as needed	HIF-1α protein *VEGF*, *GLUT1* mRNA	[81]
**hESCs** **and/or hiPSCs**	5% O_2_	6 days (primed) or 12 days (continuous)	hPSCs plated onto collagen IV-coated plates and cultured in a differentiation medium composed of α-MEM, FBS (10%) and BME (0.1 mM) Primed: cells attached in normoxic conditions for 4 hours and then subjected to hypoxia. On day 6, differentiated cells were collected, seeded on collagen-type-IV-coated plates in ECGM supplemented with FBS (2%) VEGF (50 ng/mL) and SB431542 (10 µM) for an additional 6 days Continuous: cells attached for 4 hours in normoxic conditions, and then subjected to continuous 5% O_2_ conditions	After 6 days: *CD34*, *VEGFR2*, *CD56* mRNA Primed and continuous: *CD144*, *PECAM1* mRNA; PECAM1^+^ cells; lectin binding, uptake of acLDL, tube formation on Matrigel Continuous: endothelial-like morphology with bundles of elongated cells and cobblestone area-forming cells; CD144^+^ cells; CD144 and PDGFRβ localized with CD144^+^ clusters surrounded by PDGFRβ^+^ cells	[82]
**hESCs and/or hiPSCs**	1% and 5% O_2_	up to 3 days	hESC and hiPSC cells grown on an inactivated mouse embryonic feeder layer in a growth medium consisting of 80% ES-DMEM/F12 supplemented with 20% KSR and FGF2 (4 and 10 ng/mL for hESCs and hiPSCs, respectively). For the experiment, cells were seeded on Matrigel-coated plates for feeder-free culturing in a conditioned medium supplemented with the same FGF2 concentrations above. Cells were allowed to attach in atmospheric oxygen for 24 h before the culture under low oxygen tension.	HIF-1α protein *VEGF*, *ANG1*, *ANG2*, *GLUT1* mRNA	[88]

List of abbreviations: acLDL—acetylated low-density lipoprotein; ACTA2—actin alpha 2, also known as alpha smooth muscle actin, α-SMA; ADM—adrenomedullin; ANG1—angiopoietin 1; ANG2—angiopoietin 2; ANGPTL4—angiopoietin-like 4; α-MEM—Minimum Essential Medium Eagle-alpha modification; B27—serum-free supplement used to support the growth and viability; BME—β-mercaptoethanol; BMP4—bone morphogenetic protein 4; BSA—bovine serum albumin; CD144—cluster of differentiation 144, also known as vascular endothelial cadherin, VE-cad; CD34—cluster of differentiation 34, transmembrane phosphoglycoprotein; CD56—cluster of differentiation 56, also known as neural cell adhesion molecule, NCAM; CHIR—specific inhibitor of glycogen synthase-3β; DLL4—delta-like 4; DMEM—Dulbecco’s Modified Eagle Medium; DMEM/F-12—Dulbecco’s Modified Eagle Medium/Nutrient Mixture F-12; ECDM—Endothelial Cell Differentiation Medium; ECGM—endothelial cell growth media; ECM—extracellular matrix; ECs—endothelial cells; EGM2—Endothelial Cell Growth Medium 2; EPHB2—ephrin receptor B2; EPO—erythropoietin; FBS—fetal bovine serum; FGF2—basic fibroblast growth factor, bGFG; FGFR1—fibroblast growth factor receptor 1; GLUT1—glucose transporter 1; hEBs—human embryoid bodies; hESCs—human embryonic stem cells; HIF-1α—hypoxia-inducible factor-1α; HIF-2α—hypoxia-inducible factor-2α; hiPSCs—human induced pluripotent stem cells; hPSCs—human pluripotent stem cells; IGF-1—insulin-like growth factor-1; KSR—knockout serum replacement; LDHA—lactate dehydrogenase A; mEBs—mouse embryoid bodies; mESCs—mouse embryonic stem cells; MTG—monothioglycerol; mVEGFR1—membrane-bound vascular endothelial growth factor receptor 1; N2—serum-free supplement based on Bottenstein’s N-1 formulation; NEAA—nonessential amino acids; NOTCH1—Notch homolog 1, translocation-associated; PDGFB/PDGF-BB—platelet-derived growth-factor beta/platelet-derived growth factor beta polypeptide; PDGFBR—platelet-derived growth factor receptor beta; PDK1/3/4—pyruvate dehydrogenase kinase 1/3/4; PECAM1—platelet endothelial cell adhesion molecule, also known as cluster of differentiation 31, CD31; SB431542—inhibitor of the activin receptor-like kinase (ALK) receptors, ALK5, ALK4 and ALK; SDF-1—stromal cell-derived factor-1; sVEGFR1—soluble vascular endothelial growth factor receptor 1; TIE1—tyrosine kinase with immunoglobulin-like and EGF-like domains 1; TIE2—tyrosine kinase with immunoglobulin-like and EGF-like domains 2; VCAM-1—vascular cell adhesion protein-1; VEGF—vascular endothelial growth factor A; VEGFB—vascular endothelial growth factor B; VEGFR1—vascular endothelial growth factor receptor 1; VEGFR2—vascular endothelial growth factor receptor 2; vWF—von Willebrand factor. ^#^, not all analyses were performed at the endpoint of the experiment; * not directly compared to normoxia.
